# Emergency Surgery for Left Atrial Angiosarcoma Presenting with Acute Respiratory Failure: A Fatal Case Report

**DOI:** 10.70352/scrj.cr.25-0759

**Published:** 2026-05-02

**Authors:** Anna Tsuji, Kenji Suzuki, Motohiro Maeda, Takahiro Tachibana, Eitaro Kodani, Norio Motoda, Shun-Ichiro Sakamoto, Yosuke Ishii

**Affiliations:** 1 Department of Cardiovascular Surgery, Nippon Medical School Musashikosugi Hospital, Kawasaki, Kanagawa, Japan; 2 Department of Cardiovascular Medicine, Nippon Medical School Tama Nagayama Hospital, Tama, Tokyo, Japan; 3 Department of Diagnostic Pathology, Nippon Medical School Musashikosugi Hospital, Kawasaki, Kanagawa, Japan; 4 Department of Cardiovascular Surgery, Nippon Medical School Hospital, Tokyo, Japan

**Keywords:** cardiac tumor, angiosarcoma, surgical resection

## Abstract

**INTRODUCTION:**

Primary cardiac angiosarcoma is an extremely rare and aggressive malignancy with a poor prognosis. Herein, we describe a case of left atrial angiosarcoma presenting with acute respiratory failure that required emergency surgical intervention, highlighting the condition’s diagnostic and therapeutic challenges.

**CASE PRESENTATION:**

On routine chest radiography, a mass shadow in the right lower lung field was detected in a 75-year-old male, initially suspected to be lung cancer or a pulmonary abscess. During hospitalization for diagnostic workup, the patient’s respiratory function suddenly deteriorated upon mobilization. In addition, contrast-enhanced CT revealed a large intracardiac mass occupying the left atrium. Following emergent intubation for severe hypoxemia, he was transferred to our institution for surgical intervention. Laboratory findings showed elevated inflammatory markers (white blood cells, 22850/μL; C-reactive protein, 15.9 mg/dL), coagulopathy (D-dimer, 18.67 μg/mL), and severe metabolic acidosis (pH 7.15; lactate, 31 mmol/L). Transesophageal echocardiography confirmed a large mass filling the left atrium, nearly incarcerated in the mitral valve orifice. Subsequently, emergency surgery was performed under cardiopulmonary bypass. Through a right atrial and transseptal approach, the friable tumor filling the left atrium was removed. However, the right lower pulmonary vein inlet showed extensive tumor involvement with residual attachment. Despite extensive resection and thorough cavity irrigation, complete resection could not be definitively confirmed. Initially, the patient regained consciousness without neurological deficits and was successfully weaned from mechanical ventilation on POD 6. However, severe tachypnea and copious bloody sputum production developed on POD 7, necessitating reintubation and tracheostomy. Despite hemodynamic stability, he experienced progressive type II respiratory failure, which caused his death on POD 14. Histopathological analysis revealed positivity for vascular endothelial markers (CD31, ERG, factor VIII) and absence of MDM2 expression, confirming the diagnosis of angiosarcoma. Pulmonary hemorrhagic necrosis from tumor infiltration or pulmonary tumor thrombotic microangiopathy might have caused his rapid respiratory deterioration.

**CONCLUSIONS:**

Left atrial angiosarcoma can present with acute life-threatening complications requiring emergency surgery. Despite successful tumor debulking, the prognosis remains poor because of aggressive tumor biology and potential pulmonary metastases. This case emphasizes that management of primary cardiac angiosarcoma remains challenging despite prompt surgical intervention.

## Abbreviations

PT/INR
: prothrombin time with an internationalized normal ratio
TEE
: transesophageal echocardiography
WBC
: white blood cell

## INTRODUCTION

Primary cardiac tumors are rare (<0.33% of all tumors), with approximately 25% of them being malignant.^1,2)^ Among the histological subtypes of primary malignant cardiac tumors, cardiac angiosarcoma is the most common.^3)^ This malignant type progresses rapidly and is often diagnosed at an advanced stage, frequently with pulmonary or systemic metastases.^3)^ In this report, we present a rare case of left atrial angiosarcoma requiring emergency surgery due to acute respiratory failure, and discuss the diagnostic and therapeutic challenges encountered, supported by a review of the literature.

## CASE PRESENTATION

The patient was a 75-year-old man with a history of old myocardial infarction who was under the care of another physician. Although not noted on a chest radiograph taken 7 months prior, a chest radiography for routine health screening 1 month before admission revealed a mass shadow in the right lower lung field. Lung cancer or a pulmonary abscess was suspected; thus, he was admitted to a previous hospital for further evaluation. However, before undergoing bronchoscopy for definitive diagnosis, he experienced sudden respiratory deterioration upon mobilization on the 9th day of hospitalization. No echocardiography had been performed prior to this. Echocardiography after the worsening of respiratory status showed an ejection fraction of approximately 40% with reduced anterior wall motion, but no significant change from previous findings. Contrast-enhanced CT revealed a large intracardiac mass occupying the left atrium.

Owing to severe hypoxemia, he was intubated and emergently transferred to our institution for surgical intervention.

**Fig. 1:**
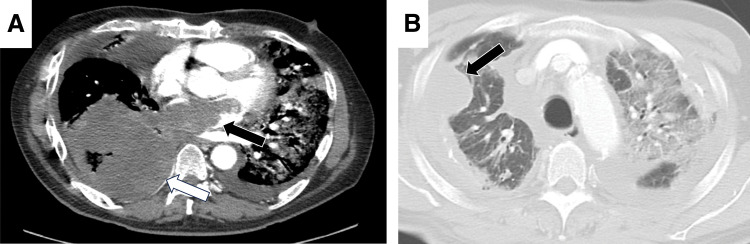
(**A**) Preoperative contrast-enhanced CT reveals a large mass in the right lung field (white arrow) and a lesion filling the left atrium (black arrow). (**B**) Preoperative CT using lung window setting demonstrates a wedge-shaped mass in the right upper lung field (black arrow) in addition to the pulmonary lesion contiguous with the left atrium.

**Fig. 2:**
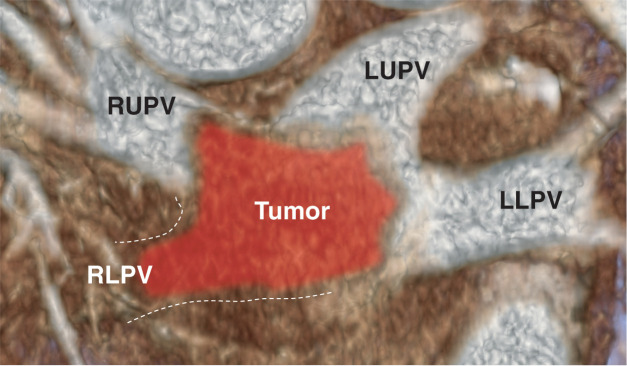
A schema based on the coronal slice of the preoperative contrast-enhanced CT is shown. A tumor filling the left atrium is observed, and the right lower pulmonary vein is completely obstructed. LLPV, left lower pulmonary vein; LUPV, left upper pulmonary vein; RLPV, right lower pulmonary vein; RUPV, right upper pulmonary vein

**Fig. 3:**
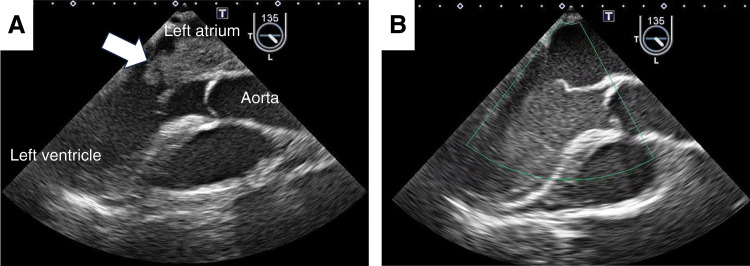
(**A**) Preoperative TEE shows a mass occupying the entire left atrial cavity (white arrow), with tumor entrapment at the mitral valve. (**B**) The tumor was no longer detected within the visual field of postoperative TEE. TEE, transesophageal echocardiography

**Table 1** shows laboratory test results on admission. Furthermore, post-intubation arterial blood gas analysis showed carbon dioxide retention with severe metabolic acidosis (pH, 7.15; HCO_3_⁻, 25.7 mmol/L; lactate, 31 mmol/L) due to low cardiac output. Chest radiography revealed diffuse bilateral decreased translucency. Contrast-enhanced CT conducted at our institution also detected the intracardiac mass occupying the left atrium, as well as a large mass in the right lung (**Fig. 1A**). The schema is shown in **Fig. 2**. Preoperative TEE further confirmed the large mass filling the left atrium, nearly incarcerated in the mitral valve orifice (**Fig. 3A**). Based on the clinical course at the previous hospital, metastasis of the pulmonary tumor or left atrial invasion was suspected. However, the diagnostic workup was still in progress, and a definitive diagnosis had not been established; therefore, the prognosis remained uncertain. In the present case, palliative resection of the left atrial tumor was performed for life-saving purposes.

**Table 1:** Laboratory test results on admission

WBC count	22850 μL
Hemoglobin	11.2 g/dL
Hematocrit	37.3%
Creatinine	1.22 mg/dL
Lactate dehydrogenase	669 U/L
C-reactive protein	15.9 mg/dL
N-terminal pro-brain natriuretic peptide	3785 pg/dL
PT/INR	1.36
aPTT	41.2 s
Fibrinogen quantity	607.6 mg/dL
D-dimer	18.67 μg/mL
Carcinoembryonic antigen	1.8 ng/mL
Neuron-specific enolase	48.8
Cytokeratin 19 fragment	4.4
Sialyl-Lewis^X^	31.8 U/mL
Progastrin-releasing peptide	11.7 pg/mL
Squamous cell carcinoma–related antigen	1.7 mg/mL

aPTT, activated partial thromboplastin time; PT/INR, prothrombin time with an international normalized ratio; WBC, white blood cell

**Fig. 4:**
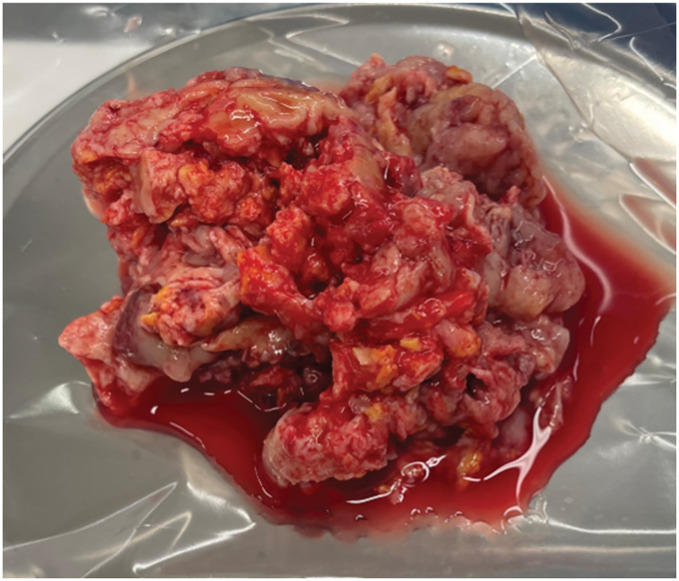
Friable tumor tissue fills the left atrial cavity.

Emergency surgery was then performed under cardiopulmonary bypass established via ascending aortic perfusion and bicaval drainage. After the right atrium was opened, the atrial septum was incised to access the left atrium. A friable tumor occupying the entire atrial cavity was noted and removed using forceps and suction. Subsequently, no residual tumor was observed macroscopically around the orifices of the left upper, left lower, and right upper pulmonary veins or the mitral valve. Meanwhile, the right lower pulmonary vein inlet was occupied by tumor tissue with residual attachment, from which as much tumor as possible was resected (**Fig. 4**). Thereafter, the cavity was thoroughly irrigated with saline, followed by pressure ventilation by the anesthesiology team, facilitating tumor expulsion from the pulmonary veins.

Following confirmation of the absence of significant mitral regurgitation, both the atrial septum and right atrium were closed. The surgery was completed after the patient was weaned from cardiopulmonary bypass uneventfully. Postoperative TEE confirmed that the intracardiac mass was completely resected (**Fig. 3B**).

Postoperatively, the patient regained consciousness without signs of limb paralysis. Despite the development of transient arrhythmia and acute liver dysfunction, which were managed with continuous hemodiafiltration and temporary pacing, no overt heart failure occurred.

Furthermore, respiratory function gradually improved; thus, on POD 6, the patient was extubated, with hemodynamic stability maintained without catecholamine support. However, on the following day, severe tachypnea and copious sputum production developed despite ongoing oxygen therapy since extubation. His chest radiograph revealed diffuse infiltration in the left lung. Laboratory test results were as follows: WBC count, 18140/μL; C-reactive protein, 11.5 mg/dL; hemoglobin, 11.5 g/dL; hematocrit, 36.3%; creatinine, 1.00 mg/dL; lactate dehydrogenase, 655U/L; PT/INR, 1.60; activated partial thromboplastin time, 35.2 s; and fibrinogen quantity, 292.5 mg/dL. Additionally, arterial blood gas analysis under O_2_ 10 L reservoir mask was as follows: pH 7.477; pCO_2_, 36.6 mmHg; PO_2_, 69.7 mmHg; HCO_3_⁻, 26.7 mmol/L; base excess, 3.5 mEq/L; lactate, 20 mmol/L. Administration of 10 L/min oxygen via a reservoir mask corresponds to an FiO_2_ of approximately 0.9, yielding a PaO_2_/FiO_2_ ratio of 77.4, which indicates severe hypoxemia. Considering these respiratory difficulties, on POD 7, the patient underwent reintubation and tracheostomy, along with bronchoscopy, which showed frothy, blood-tinged sputum.

Despite hemodynamic stability, respiratory acidosis caused by progressive type II respiratory failure worsened, leading to death on POD 14.

**Fig. 5:**
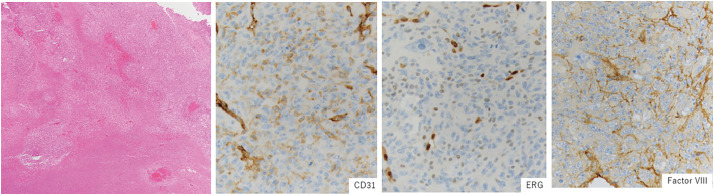
Histology reveals a largely necrotic tumor with focal viable areas and hemorrhage. Immunohistochemical staining was positive for vascular endothelial markers (CD31, ERG, and factor VIII).

Histopathological examination of the cardiac tumor revealed confluent necrosis and atypical epithelioid and spindle cell proliferation with prominent nucleoli. The tumor cells were positive for vascular endothelial markers (CD31, ERG, and factor VIII) (**Fig. 5**), in addition to AE1/AE3, vimentin, and α-smooth muscle actin, and negative for other markers (CK7, CK20, p40, TTF-1, desmin, h-caldesmon, S-100, myogenin, MyoD1, MDM2, and CDK4) by immunohistochemistry. Ki-67 staining was positive, showing a high proliferation rate (70%–80%). These findings were confirmed by a diagnosis of angiosarcoma.

## DISCUSSION

Immunohistochemistry of the resected tumor showed no MDM2 expression, suggesting that intimal sarcoma was unlikely.^4)^ Most of the findings supported the diagnosis of angiosarcoma.^5,6)^

In cases of cardiac angiosarcoma presenting with circulatory failure, emergent tumor resection may be required, with the definitive diagnosis being established postoperatively. There have been reports of survivors following emergent surgery combined with adjuvant chemotherapy and/or radiotherapy.^7,8)^ Yin et al. identified tumor resection followed by chemotherapy as favorable prognostic factors in patients with cardiac angiosarcoma.^9)^ In the present case, chemotherapy was planned to be initiated upon recovery of the patient’s general condition following surgery. Although advanced age has been reported as an adverse prognostic factor, the patient in this case was 75 years old, which is relatively elderly.

Surgical resection is the mainstay of treatment for primary cardiac sarcomas; however, even in cases of macroscopically complete resection, the 1-year survival rate is only approximately 60%.^10)^ In our case, although all visible tumor tissue was resected intraoperatively, the patient’s condition remained unstable postoperatively. Additionally, follow-up imaging could not be performed. Therefore, we could not confirm complete resection of the tumor.

Approximately 50% of patients undergoing surgery for primary cardiac sarcoma experience postoperative complications, with 10% developing prolonged respiratory failure and 13% dying during the perioperative period.^10)^ Following extubation, our patient subsequently developed persistent respiratory failure and ultimately died on POD 14.

The patient’s rapidly progressing respiratory failure observed after POD 7 may be explained by several factors. First, tumor infiltration could cause pulmonary hemorrhagic necrosis, resulting in rapid postoperative respiratory failure. Bronchoscopy at reintubation on POD 7 revealed frothy, blood-tinged sputum, which suggests pulmonary hemorrhagic necrosis. The rapid respiratory failure may have been caused by tumor metastasis to the lungs. A previous report described a similar clinical course wherein pulmonary hemorrhagic necrosis caused by tumor infiltration was identified at autopsy,^11)^ suggesting a comparable pathophysiology to our case. Second, the rapid respiratory deterioration may have been caused by pulmonary tumor thrombotic microangiopathy, a condition typically resulting from multiple tumor metastases to pulmonary arteriolar walls. This event leads to fibrous proliferation of the vascular endothelium and localized thrombus formation, causing vascular lumen narrowing or occlusion. It is known to rapidly progress, presenting with pulmonary hypertension and right-sided heart failure.^12)^ In our case, despite the respiratory failure onset, hemodynamics remained stable, and no findings suggested pulmonary hypertension or right-sided heart failure. However, cases of pulmonary tumor thrombotic microangiopathy without such findings have been reported. Tumor lysis syndrome was also considered, but it was eventually ruled out because of the absence of electrolyte abnormalities or renal dysfunction.

Furthermore, the left atrial cavity was filled with tumor tissue, extensively involving the orifice of the right lower pulmonary vein, suggesting a possible direct extension into the pulmonary mass observed on CT. In addition to the pulmonary lesion contiguous with the left atrium, a wedge-shaped mass in the right upper lung field was detected on preoperative CT taken on the day of surgery (**Fig. 1B**). No autopsy was performed; thus, this pulmonary mass could not be further examined, and its malignancy remains uncertain. However, this finding may suggest metastasis via the pulmonary veins. Primary cardiac angiosarcoma most commonly metastasizes to the lungs, accounting for 17.2% of cases.

## CONCLUSIONS

Left atrial angiosarcoma can present with acute lethal complications that necessitate emergency surgery. Following successful tumor debulking, poor prognosis may persist because of the tumor’s aggressive nature and potential pulmonary metastases. Therefore, despite timely surgical intervention, primary cardiac angiosarcoma remains challenging to manage.

## DECLARATIONS

### Availability of data and materials

The datasets supporting the conclusions of this article are included within the article.
